# Implementing the Family-Led Care model for preterm and low birth weight newborns in Malawi: Experience of healthcare workers

**DOI:** 10.4102/phcfm.v12i1.2266

**Published:** 2020-08-17

**Authors:** Patani Mhango, Effie Chipeta, Adamson S. Muula, Judith Robb-McCord, Patrice M. White, James A. Litch, Irene Kamanga, Rebecca Freeman, Anne-Marie Bergh

**Affiliations:** 1 Centre for Reproductive Health, College of Medicine, University of Malawi, Blantyre, Malawi; 2 Department of Public Health and Epidemiology, College of Medicine, University of Malawi, Blantyre, Malawi; 3 Project Concern International, San Diego, and Washington, DC, United States of America; 4 American College of Nurse-Midwives, Silver Spring, Maryland, United States of America; 5 Global Alliance to Prevent Prematurity and Stillbirth, Seattle, United States of America; 6 Project Concern International, Zomba, Malawi; 7 UP-SAMRC Unit for Maternal and Infant Health Care Strategies, Faculty of Health Sciences, University of Pretoria, Pretoria, South Africa

**Keywords:** preterm birth, low birth weight, neonates, family-centred care, kangaroo mother care, quality of care, healthcare providers, Malawi

## Abstract

**Background:**

Every Preemie–SCALE developed and piloted the Family-Led Care model, an innovative, locally developed model of care for preterm and low birth weight babies receiving kangaroo mother care.

**Aim:**

The aim of this study was to describe healthcare workers’ experience using Family-Led Care.

**Setting:**

This study was conducted in five health facilities and their catchment areas in Balaka district, Malawi.

**Methods:**

The mixed-methods design, with two data collection periods, included record reviews, observations and questionnaires for facility staff and qualitative interviews with health workers of these facilities and their catchment areas. The total convenience sample comprised 123 health professionals, support staff and non-professional community health workers.

**Results:**

Facility-based staff generally had positive perceptions of Family-Led Care (83%). Knowledge and application-of-knowledge scores were 69% and 52%, respectively. A major change between the first and the second data periods was improvement in client record-keeping. Documentation of newborn vital signs increased from 62% to 92%. Themes emerging from the qualitative interview analysis were the following: benefits of Family-Led Care; activities supporting the implementation of Family-Led Care; own care practices; and families’ reaction to and experience of Family-Led Care.

**Conclusion:**

This article reports improved quality of care through better documentation and better follow-up of preterm and low birth weight babies receiving kangaroo mother care according to the Family-Led Care model. Overall, health workers were positive about their involvement, and they reported positive reactions from families. Lessons learned have been incorporated into a universal Family-Led Care package that is available for adaptation by other countries.

## Background

In the last decade, three landmark reports were published on preterm birth (PTB) and stillbirth: *Global report on preterm birth and stillbirth: The foundation for innovative solutions and improved outcomes* (2010),^1^ *Born Too Soon: The global action report on preterm birth* (2012)^2^ and *Every Newborn: An action plan to end preventable deaths* (2014).^3^ Since their publication, there has been increased interest in and focus on the prevention of PTB, the care of the preterm newborn and treating complications associated with PTB and low birth weight (LBW).^4^ The World Health Organization defines preterm as ‘babies born alive before 37 weeks of pregnancy are completed’^5^ and LBW as ‘weight at birth less than 2500 g.’^6^

In 2016, PTB complications were estimated to be the leading cause of all deaths in children under 5 years of age worldwide (18%).^7^ A global health systems bottleneck analysis found that some of the biggest obstacles to scaling up essential interventions to reduce neonatal deaths were associated with the care of small and sick newborns, for example, inpatient supportive care for these newborns, the management of severe infections and kangaroo mother care (KMC).^8^

Every Preemie–SCALE (Every Preemie), a consortium of Project Concern International, the Global Alliance for the Prevention of Preterm Birth and Stillbirth and the American College of Nurse Midwives, was a 5-year project that ran from 2014 to 2019 and was supported by the United States Agency for International Development (USAID). The ultimate aim of the project was to influence global and country-level dialogue and action for preterm and LBW newborns. Malawi is one of 25 countries prioritised by USAID for maternal and child health support and was selected as an Every Preemie demonstration country for targeted technical assistance. The project designed, implemented and tested the Family-Led Care model for resource-constrained environments with limited human resources. Lessons learned were to be used to strengthen the model of care and adapt it for expanded use in Malawi and other countries.

Malawi is one of the poorest countries in the world and was ranked 171 out of 188 countries and territories on the human development index (0.477) in 2017.^9^ The estimated PTB incidence in Malawi is currently 10.5% and the LBW rate is 14%.^10^ Although Malawi was one of few countries to achieve its Millennium Development Goal 4, quality of newborn care, especially the provision of routine KMC for preterm and LBW babies, was identified as an area of focus to further decrease under-five mortality.^11,12^ The Government of Malawi is committed to improving the care of maternal, newborn and child health services and to strengthening community-based healthcare.^13^ More than 90% of deliveries in Malawi take place in a health facility.^14^

### Aim and objectives of the study

This implementation research was undertaken to gather evidence on the real-life implementation of the newly developed Family-Led Care model in Malawi. In this article, we focus on service delivery. A second study focusing on family caregivers will be reported elsewhere.

The overall aim of this study was to describe the experience of facility-based health professionals and support staff and community-based health surveillance assistants (HSAs) in delivering Family-Led Care in the Balaka district in Malawi. Specific objectives included exploring the following: knowledge of preterm and LBW identification for admission to a health facility, referral and discharge; adherence to clinical care standards; competence in providing inpatient and follow-up care for preterm and LBW newborns; attitudes towards Family-Led Care and associated support; and health worker perceptions of mothers’ and families’ reaction to Family-Led Care.

### Description of the Family-Led Care model

The Every Preemie project in Malawi began in 2015–2016 with a series of field visits and stakeholder consultations with the Ministry of Health, USAID Malawi, and local partners to identify country needs in the provision of care around PTB and LBW. The Balaka district in southern Malawi was identified as the target district for project engagement. This district covers an area of 2142 km² and had a population of 438 379 in 2018.^15^ The population of women of childbearing age was 91 176 in 2017, and the number of expected pregnancies and deliveries per annum was approximately 18 592 (information provided by the Balaka District Health Office).

The development of the Family-Led Care model was a collaborative effort primarily between Every Preemie and the Balaka District Health Management Team. The aim was to design a model:

- to improve the quality of care provided in KMC units or corners by strengthening KMC practices for preterm and LBW babies in health facilities according to the Malawi KMC guidelines^16^
- to engage parents and other family members in the care of their newborns in the health facility and at home post-discharge
- to ensure a continuum of care from facility to household.

### Components of the Family-Led Care model

**FIGURE 1:**
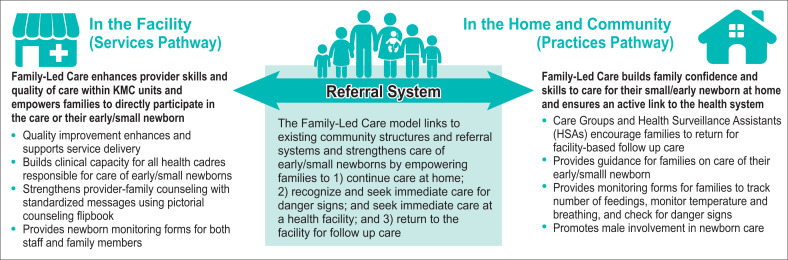
Graphic depiction of the Family-Led Care model.^10^

The Family-Led Care model positions families as active, confident participants in the care of their preterm and LBW babies in the health facility and at home. Family members are the central role-players that constitute the continuum between community and healthcare facility and lead the health-seeking behaviour in antenatal and postnatal care. The model promotes improved quality of care at the facility level and increased access to and utilisation of care through a functional referral system to address morbidity and mortality of preterm and LBW babies after discharge from the health facility. Figure 1^10^ depicts the conceptual model of the three interlinked components of Family-Led Care and illustrates how it should be implemented. Three main groups of people are involved in implementation:

- caregivers of preterm and LBW babies in the health facility and at home (mothers, fathers and other family members)
- facility-based healthcare staff including professionals (nurse-midwives, medical assistants and clinical officers) and support staff (hospital attendants)
- community-based health agents including community health workers (HSAs) and community volunteers (e.g. care groups).

Resource materials were developed to accompany the implementation of Family-Led Care (https://www.everypreemie.org/malawi/family-led-care/). The counselling flipbook has standardised messages that mothers and families receive in the delivery room or on admission to the KMC room/space and before discharge from the health facility. Parents also receive a take-home leaflet with basic reminder messages to share with other family members. Other important resources include monitoring forms and checklists. Mothers or other family members tick the basic care family monitoring form after each feed and twice per day when they have checked for danger signs. Healthcare providers document twice-daily assessments of all small babies on a basic KMC monitoring form. In addition, 2- and 3-h feeding charts were adapted from existing government forms in use.

### Implementation process

The Family-Led Care model was launched at nine healthcare facilities and their catchment areas in the Balaka district in January 2017. The facilities included the district hospital, five health centres with delivery and inpatient KMC services and three centres with follow-up care services only. Table 1 contains a summary of all the strategies and activities involved in the implementation of the Family-Led Care model.

**TABLE 1:** Family-Led Care implementation strategies and activities.

Strategy or activity
**In the Health Facility – The services pathway (clinical care)** **Training and capacity building** (9 facilities; 180 staff) Healthcare providers (*n* = 124; 5 days total) ■ Midwives, nurses, clinical officers, medical assistants ■ Training package: Essential Care for Every Baby and Essential Care for Small Babies (3 days), Family-Led Care (2 days) Support staff (*n* = 56; 2.5 days) ■ Mostly hospital attendants ■ Trained in the basics of Family-Led Care **Infrastructure, equipment and supplies** Locally procured calibrated feeding cups Weighing scales with proper tolerances Baby wrappers, hats and booties Digital thermometers Assorted plastic buckets (to decontaminate feeding cups and promote hand washing) Electric space heaters Ensured all facilities had a KMC corner or room Upgrades to the KMC room or corner to create a more appealing environment for families (e.g. window curtains, bed wedges, mothers’ gowns) **Supervision** Nomination of a district-level KMC coordinator Monthly supervision visits to all health facilities in district Quarterly review meetings **Quality improvement (QI)** Process mapping Training of 12 QI mentors – one mentor each for eight health centres and two for the hospital and one of the bigger health centres Each facility: QI team with leader (max. 12 members) – decide on own projects Monthly coaching visits to each health facility – focus on record-keeping, data quality and completeness, linkage with community health workers Monthly mentors’ meeting – sharing of experiences and challenges Five collaborative learning sessions: ■ Opportunity for each facility to showcase its project(s) and progress with implementation ■ Last meeting: QI sustainability commitments for different stakeholders mapped out Dashboard with progress and outcome indicators kept
**Strengthening the referral system and follow-up care** Referral forms (completed with a follow-up date) developed KMC registers used Diary to book babies for follow-up implemented Families prepared for follow-up Standardised content of follow-up visit Tracking of babies lost to follow-up
**At home and in the community – The practices pathway (community engagement)** **Take-home materials for families** Take-home leaflet with basic messages Basic care family monitoring form to complete **Orientation in Family-Led Care** Combined with training and orientation in community newborn care modules Health surveillance assistants (community health workers) (*n* = 93) Health and nutrition promoters (*n* = 46) Community volunteers (care group mothers) (*n* = 2333) **Community sensitisation** Community awareness meetings

KMC, kangaroo mother care; QI, quality improvement.

## Methods

**FIGURE 2:**
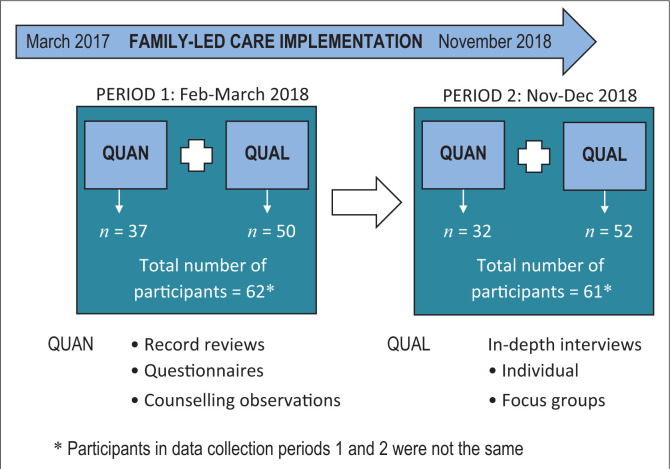
Mixed-methods research design.

A mixed-methods approach with two data collection points was implemented in 2018 (henceforth called ‘data periods’ 1 and 2). Figure 2 provides an overview of the research design.

### Study setting and sample

The study was conducted in five of the six Family-Led Care health facilities with maternity services in Balaka district. The sixth facility was used to pilot test and refine the tools before beginning the study.

Study participants were healthcare staff at these facilities with training in Family-Led Care. They included professional providers (nurse-midwives, clinical officers, medical assistants) and facility support staff (hospital attendants) working in the labour and delivery ward, postnatal care ward and/or nursery and KMC unit/corner. HSAs who were working in the community catchment areas of these five facilities and who had received orientation in Family-Led Care were also recruited. Facility staff and HSAs with under 3 months’ experience in the facility or catchment area were excluded from participation.

Implementation of the model was a dynamic process, and additional staff were trained over the period of implementation. Data period 1 (February 2018 – March 2018) targeted staff that had been trained in 2016 and 2017; data period 2 (November 2018 – December 2018) focused on staff trained in August 2018. This enabled us to gather data and perspectives from more participants to inform the understanding of implementation over time and what would be needed to further scale up the Family-Led Care model.

Convenience sampling was used in all data collection activities in both data periods, depending on which facility staff members and HSAs were available at the time of data collection. Because data collectors were moving between the sites, they missed some of the trained staff. Furthermore, some staff were unavailable because they were on night duty, leave or other assignments away from their facilities during the data collection periods. Table 2 lists the cadres of recruited health workers for the different data collection tools (health professionals, hospital attendants and HSAs).

**TABLE 2:** Overview of data collection tools, target audiences and number of participants.

Types of tools	Method	Target audience			Number of records/participants		
		HP	HA	HSA	Data period 1	Data period 2	Total
**Retrospective record reviews**							
1. Number of babies born < 2500 g	Data sheet				212	243	455
2. Knowledge of and adherence to admission and discharge criteria	Data sheet				27	50	77
3. Adherence to monitoring	Data sheet				27	50	77
4. Follow-up care adherence	Data sheet				27	50	77
**Counselling observations**							
5. Initiating basic care for preterm and LBW babies	Checklist	✓	✓		8	12	20
6. Pre-discharge counselling for home care	Checklist	✓	✓		7	11	18
7. Follow-up care	Checklist	✓	✓		8	13	21
**Questionnaires**							
8. Referral knowledge assessment	Open-ended question	✓			19	14	33
9. Family-Led Care knowledge assessment	10 true–false questions	✓			19	14	33
10. Case studies (knowledge application/skills)	Case studies†	✓			18	15	23
11. Staff perceptions of Family-Led Care	8-item Likert scale	✓	✓		27	24	51
**Interviews**							
12. Staff perceptions of Family-Led Care	Individual interviews	✓	✓		15	6	21
13. Staff perceptions of Family-Led Care	Focus groups	✓	✓		5‡	17§	22
14. HSAs’ perceptions of PTB, LBW and Family-Led Care	Focus groups			✓	25§	29¶	54

† , Random selection of four of eight case studies.

‡ , One focus group.

§ , Four focus groups.

¶ , Five focus groups.

HP, health professionals (nurse-midwives, clinical officers, medical assistants); HA, hospital attendants (support staff); HSA, health surveillance assistants; PTB, preterm birth; LBW, low birth weight.

### Data collection

Different types of data collection tools were developed to cover all the objectives of the study (see Table 2 for details). Both data collection periods used the same set of tools. Three data sheet templates pertaining to admission and discharge, in-facility monitoring and follow-up, respectively, were used to record data from 3-month retrospective record reviews (convenience sample of at least 10 records per facility per data period). The tools for facility-based professionals and support staff included three counselling observation checklists and a Likert-scale questionnaire in English and Chichewa to probe attitudes towards and perceptions of Family-Led Care. Three questionnaires in English targeted health professionals only, namely, an open-ended question on referral, true–false knowledge questions and case studies. English and Chichewa versions of the interview guides were available for individual and focus group interviews with all cadres of health workers, including HSAs.

All research assistants were fluent in English and Chichewa and interviewed participants in the language of their choice. The choice between individual and group interview depended on the availability of health workers. Only focus group interviews were conducted with HSAs. All interviews were audio-recorded with the permission of participants.

### Data management and analysis

Quantitative data were entered on Microsoft Access and analysed in Stata 15 for Windows and Excel. Descriptive summary statistics were generated and results crosschecked.

All audio-recordings were transcribed, and all Chichewa transcripts were translated into English. One researcher (PM) crosschecked the translations and made some cultural and linguistic adaptations to facilitate understanding and interpretation for the intended readers and data analysts. The qualitative data analysis team met to reach consensus on emerging themes and develop a codebook. Transcript documents were imported into ATLAS.ti for further analysis. Another researcher (A-MB) established the feasibility of the outcomes of the analysis and interpretation.

### Ethical consideration

The study protocol was approved by the Malawi College of Medicine’s Research and Ethics Committee (Protocol number: P.10/17/2303) and the Institutional Review Board of Project Concern International (Protocol number: 26). Written informed consent was obtained from all health worker participants. Mothers being observed in their interaction with a health worker(s) also gave written informed consent. Mothers younger than 18 years provided assent, while their parent or legal guardian signed the consent form.

## Results

### Participant demographics

For the entire study, 123 health workers from the five health facilities and their catchment areas were recruited, 62 in the first data period and 61 in the second data period (Figure 2). Participants included clinical officers (*n* = 5), medical assistants (*n* = 2), nurse-midwives (*n* = 31), hospital attendants (*n* = 31) and HSAs (*n* = 54). Table S1 in the supplementary data file provides more detail. In data period 1, male and female participants were equal in number (*n* = 31), whereas in period 2, there were slightly more female (*n* = 34) than male participants (*n* = 27). More than half of the participants were from the district hospital (27%) and one health centre (29%) close to the town of Balaka. The rest of the participants were evenly spread across the remaining three health centres (13–16%).

### Record review

A total of 455 babies born preterm or LBW were recorded for the two 3-month periods before the data collection points, of which 238 (52%) were admitted to inpatient KMC. It is not known how many of the remaining babies were initiated on ambulatory KMC or came back for regular follow-up. Slightly more babies were recorded in the second data period (243 vs. 212). In the two periods combined, the majority of preterm and LBW babies were born in the district hospital (*n* = 378) compared to 89 in all four health centres combined. Table S2 in the supplementary data file provides more detail.

*Poor in-facility record-keeping* was identified as a problem in the first round of record reviews, with some health facilities having no or very few case notes or register entries to review. Many available records were incomplete. Research assistants were therefore unable to achieve the proposed sample of 10 records per facility or 50 records in total. This is reflected in Table 2, which shows that only 27 records could be reviewed in the first period. Of these 27 records, only 19 had birth and discharge dates recorded. During the second data collection period, the complete sample of documents was available for review.

*Adherence to standards of care* was measured in terms of proper record-keeping, regular monitoring of the baby, following discharge criteria and providing quality follow-up care. With regard to *regular monitoring of babies’ vital signs, feeding and weight*, all facilities had high adherence rates in data period 2 compared to data period 1. The mean adherence score for documenting monitoring activities improved from 62% to 92%. One health centre with no records available in data period 1 scored 100% on all observation items in data period 2. The district hospital improved from 34% to 91%. The health centre administered by the Christian Medical Association of Malawi had high documentation adherence rates of ≥ 80% in both data periods. Table S3 in the supplementary data file provides more details.

*Adherence to discharge criteria* was measured according to some of the country KMC guidelines: absence of danger signs, birth weight regained, and a discharge weight of > 1500 g for the district hospital and > 1800 g for the health centres.^16^ Less than half of babies were discharged before regaining birth weight. The district hospital discharged all but one baby at > 1500 g and the remaining 27 discharges below 1800 g occurred at the health centres (20% of health centre discharges). There was no difference in discharge rates from data period 1 to data period 2, and there was a decrease in the percentage of babies regaining birth weight before discharge in data period 2. Table S4 in the supplementary data file provides more details.

Fewer records were available for *follow-up care* (51 out of 77), as some babies had already achieved a weight of 2500 g, at which point they were (correctly) discharged from the special KMC follow-up. Follow-up records also showed notable improvement between 34 and 57 percentage points in the completeness of documented observations with regard to date, weight and temperature entries (see Table S5 in the supplementary data file). In the second data period, most records and registers contained full information on the baby and 80% of patients received follow-up appointments.

### Knowledge and skills

Professional participants (*n* = 33) had to answer an open-ended question on the *criteria for referring a preterm or LBW baby* from a health centre to the district hospital. Responses were coded for four criteria: birth weight < 1500 g, presence of one or more danger signs, mother is very sick, and no surrogate to care for the newborn or no acceptance of KMC by surrogate or mother. Only one participant each mentioned the last two criteria. A birth weight of < 1500 g was mentioned by 13 (39%) participants and the presence of a danger sign by 19 (58%) participants. The percentage of participants mentioning the latter two (more important) referral criteria was much lower in data period 2 than in data period 1 (29% vs. 63%). Table S6 in the supplementary data file provides more information.

The same 33 professionals completed a set of 10 true–false questions to assess their *knowledge of preterm and LBW care*. Participants in the second data period had a lower score of 5 percentage points (67% vs. 72% in data period 1). The overall score was 69%. The trends in scores per question were similar for the two data periods, with the poorest scores for questions relating to normal weight gain, and the family’s role in monitoring the baby in hospital and at home. The supplementary data file contains a table on the content of the knowledge questions (Table S7), a table on health professionals’ overall performance (Table S8) and a figure on health professionals’ performance per question (Figure S1).

Eight case scenarios were grouped randomly into unique combinations of four to assess *provider competence in the application of knowledge to manage preterm and LBW babies*. Each participant completed one case study on weight gain, one on the calculation of feeding volumes and two case studies on danger signs (respiratory distress, refusal to feed, jaundice and fever). This activity was completed by 23 professionals. Participants scored an average of 52% for the case studies. The scores were relatively similar for data periods 1 and 2. The mean scores were below 50% for refusal to feed, jaundice and one of the cases related to weight gain. Figure S2 in the data supplement compares the scores for the different case studies for the two data periods.

*Counselling skills of facility-based staff* were observed using three checklists linked to the content of the Family-Led Care flipbook: initiating basic care (*n* = 20), counselling at discharge (*n* = 18) and follow-up counselling (*n* = 21). Where there were no or not enough real cases to observe, role-plays were staged. In total, there were 16 real-life observations and 43 role-plays. For all three counselling occasions and the two data periods combined, the counselling score was 79%. The score for data period 2 (83%) was higher than for data period 1 (74%). Details of the counselling observations and the scores are contained in Tables S9–S12 and Figure S3 in the supplementary data file.

### Attitudes and perceptions

**FIGURE 3:**
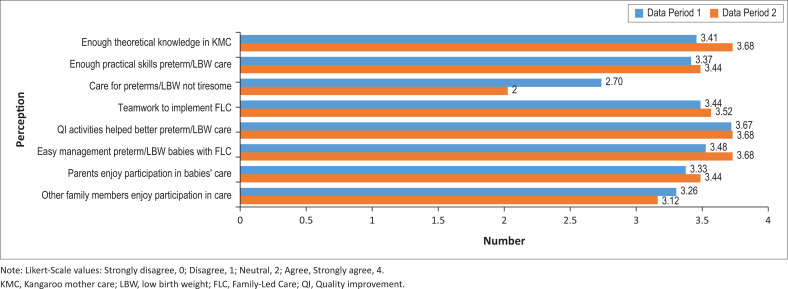
Staff perceptions of Family-Led Care.

Health workers’ attitudes towards and perceptions of Family-Led Care were probed in the in-depth individual and focus group interviews. A Likert-scale questionnaire was also completed by 51 health facility staff (professionals and hospital attendants). The overall mean score was 3.3 out of 4 (83%) for both data periods. These results were consistent across all facilities and cadres. Figure 3 contains a graphic breakdown of the scores on individual items for both data periods. The only item with a score below 3 was in response to the idea that caring for a preterm or LBW baby was tiresome (responses received an inverse value in analysis).

Subthemes and categories emerging from the qualitative interview analysis were organised around the following health worker perceptions and reports (themes): benefits of Family-Led Care, activities supporting the implementation of Family-Led Care, own preterm and LBW care practices and families’ reaction to and experience of Family-Led Care. Table 3 provides an overview of themes, subthemes and categories identified. References for direct quotations from participants start with F1, F2 and so on, which refer to the facility number; the number after the hyphen refers to the participant number.

**TABLE 3:** Healthcare workers’ perceptions of and reports on Family-Led Care.

Themes	Categories
**Benefits of Family-Led Care**	
Benefits for staff	Skills and knowledge enhanced through training
	Staff workload: increase and reduction
Benefits for preterm/LBW babies	Increased survival rates
	Better growth
Benefits for families	Better provider–parent communication
	Improved parent knowledge and confidence
	Improved family relationships and distribution of roles
	Enlightenment of men
**Activities to support Family-Led Care implementation**	
Health systems strengthening	Facility upgrades and expansions
	Resources received
	Sustainability of resources
Training	Content
	On-the-job transfer of learning
	Learning to counsel
	Improved facility–community linkage
Quality improvement	Introduction of protocols and job aids
	Improved documentation practices
**Own preterm/LBW care practices**	
Admission to KMC unit	Preterm identification
	Low birth weight identification
	Admission criteria
Referral	According to weight
	Danger signs
Health facility observation procedures	Monitoring of vital signs
	Growth monitoring
	Feeding
Repeated counselling and support to parents	Counselling with example
	Feeding
	Thermal care
	Infection prevention
Home follow-up by HSAs	Home visits – activities
**Families’ reaction to/experience of Family-Led Care**	
Acceptance of skin-to-skin care	Accepted when well explained
	Mothers’ anxiety
	Religious and cultural factors
	Adherence after discharge
Use of expressed breastmilk	For cup feeding
	For tube feeding
Family monitoring form	Completion – varying reactions
	Literacy level
Attachment	Strong mother–baby bonding
Fathers’ involvement	During hospital stay
	At follow-up
	Other support
	Traditional beliefs
KMC clothes	Mothers: opening in front
	Baby wrapper
	Babies: only partially dressed

LBW, low birth weight; KMC, kangaroo mother care; HSAs, health surveillance assistants.

With regard to the perceived benefits of Family-Led Care, health workers alluded to the observation that preterm and LBW babies were now discharged alive and thriving and that this increased their confidence and the confidence of families about caring for these newborns. They reported an improved understanding of how to involve mothers and families in participating in the safe care of their babies through ongoing counselling and support. They also appreciated the improvement in their knowledge and skills regarding preterm and LBW identification, referral, monitoring, feeding, thermal care and infection prevention. There was also reference to health workers’ ability to put their learning into practice:

‘[*W*]hen we have a baby on KMC, we … made it … routine … [*to*] monitor it regularly and the good thing is that we have charts that guide us.’ (F4-1)

‘We were missing those babies who are more than 2000 grams to 2400 grams but because of the coming of Family-Led Care, they taught us to provide the same management as to the baby who is less than 2000 grams.’ (F1-2)

Professionals acknowledged that their documentation skills had improved – ‘we are able to achieve almost 100% documentation of files of KMC babies’ (F4-1). They also discussed the effect of Family-Led Care on workload. Some felt the workload was reduced – ‘the workload is minimised a little because the family take part in taking the care of the baby’ (F4-1). Others felt Family-Led Care brought additional burdens:

‘I feel that Family-Led Care has increased our workload because babies are now followed up more from the day of discharge from a health facility. There’s a lot of things happening now like registers, monitoring tools and many more leading to the increase in workload. In the past when the mothers were counselled and went to the KMC unit, everything used to end there. But nowadays we are following up several times until when the baby is discharged from KMC.’ (F2-1)

To support the implementation of Family-Led Care, Every Preemie provided basic supplies and small upgrades to the KMC units or corners (see Table 1). Health workers appreciated the contributions that have ‘helped a lot’ (F5-1), but some mentioned sustainability as an issue – ‘so it is running out of stock’ (F1-1).

Health workers reported that families mostly accepted Family-Led Care and that mothers who had been counselled well were, with a few exceptions, happy to provide skin-to-skin care and complete the special monitoring form twice a day. For some women ‘during the first days, it is difficult to accept’ (F2-1) and literacy levels may have influenced completion of the monitoring form – ‘those that know how to read and write find it easy completing the monitoring forms’ (F2-2). Health workers also observed the positive effect of skin-to-skin care on mother–baby attachment. Although mothers were comfortable with the manual expression of breastmilk and cup feeding, they were reluctant to accept tube feeding because they believed that the tube would block the nostrils or that ‘the baby must be critically ill … about to die’ (F4-1).

Health workers mentioned that mothers found it difficult to adhere to continuous skin-to-skin care for at least 20 h a day at home in the absence of sufficient support:

‘It is tiresome because they [*mothers of preterm/LBW babies*] do not just give the child to anyone to carry unlike with a child who has normal weight … When the child has normal weight other people can carry, but when you don’t have a relative it is difficult to find another person to carry your preterm baby.’ (F3-2)

Very few fathers were reported to be involved in Family-Led Care at health facility level because the physical environment and lack of privacy in the maternity and KMC units/corners made it ‘unlikely’ that ‘they can be in the ward and put a baby on kangaroo method and be a reliable guardian’ (F1-4). After discharge from the facility, health workers reported that ‘most fathers are able to escort mothers to the hospital on the follow-up appointment dates’ (F1-3) and ‘for example, when a mother is cooking, some fathers put babies in skin-to-skin contact on the chest’ (F3-1). HSAs commented more generally that some fathers still took the traditional view that caring for babies was the responsibility of women.

## Discussion

The Family-Led Care model was designed to be embedded into the existing health system in Balaka district, Malawi. It aimed to improve the quality of care for preterm and LBW babies initiated on KMC and extend the coverage of appropriate care for these newborns. Using the lens of the World Health Organization’s health systems building blocks,^17^ the model incorporates each of the building blocks, as well as community ownership and partnership^8^ with the linkage of families and their newborns from health facility to household. The model was introduced under the *leadership* of the District Health Management Team as the body responsible for the provision of healthcare in the district. Although USAID *financing* was available for the development and implementation of the Family-Led Care model, its continuation will be part of the district’s responsibility through inclusion in the District Implementation Plan and district budgets. Additional support may be needed to train staff in district facilities not reached by the Every Preemie project.

Implementation of the Family-Led Care model included strengthening the knowledge and skills of the *health workforce* and providing basic *supplies* needed to care for preterm and LBW babies receiving KMC. Through clinical training and the quality improvement initiative, the quality of inpatient *health service delivery* and facility-based follow-up care improved and the *health information system* was strengthened via improved record-keeping. The findings from the implementation of Family-Led Care were also shared with other governance structures at national level and trainers were trained to introduce Family-Led Care in another 16 districts.

This article presents the findings of implementation research undertaken to describe the experience of healthcare workers implementing the Family-Led Care model. Theobald et al. describe the purposes of implementation research as ‘improving people’s health, informing policy design and implementation, strengthening health service delivery, and empowering communities and beneficiaries’ (p. 2214).^18^ The Family-Led Care model was designed with all these purposes in mind. The most visible achievement of the implementation of the model lies in strengthening health service delivery, which has led to participation of families (especially mothers) in the care of their preterm and LBW babies and anecdotal reports of the increased survival of preterm and LBW babies. Mothers’ and family members’ experience of Family-Led Care is the focus of a separate article submitted for publication.

### Limitations, strengths and recommendations

Because of the short time frame for implementation, it was not possible to implement a more traditional before–after research design. Furthermore, the purpose of the study was primarily to learn from the implementation process over time, hence the two data collection periods and inclusion of as many participants as possible.

Health workers stated that the training and orientation they had received and the quality improvement conducted according to individual health facility needs had enabled them to improve their record-keeping, take better care of preterm and LBW babies and assist with families’ acceptance of KMC and their empowerment to take care of their babies at home. The maternity ward, postnatal ward and KMC space lacked appropriate privacy for families, which may have limited the full uptake of Family-Led Care by fathers. This should be taken into account in any future implementation of the model, as sharing continuous skin-to-skin care at home with fathers and other family members is essential in adhering to the recommendation of at least 20 h per day.

Two other areas of service delivery that deserve special mention are staff workload and discharge criteria. Although staff reported an increased workload, the quality of care also increased. Implementation of the Family-Led Care model resulted in delivery of care for babies receiving KMC that was closer to international care standards as described in the Essential Care for Small Babies^19^ and the World Health Organization’s KMC guide.^20^ With regard to adherence to discharge criteria, the data collection tool did not make provision for sufficient discrimination between the finer nuances in the discharge criteria listed in the Malawi KMC guidelines.^16^ The tool did not provide for linking the criterion of regaining birth weight with the actual birth weight of a baby. For example, according to the guidelines, babies with a birth weight above 1800 g could be discharged before regaining birth weight. The 55% of babies discharged before regaining birth weight could have been in the > 1800 g birth weight category.

When considering the implementation of the Family-Led Care model, it may be helpful for policy-makers and implementers to revisit the country’s KMC guidelines and other preterm and LBW care documents to confirm the clarity and completeness of criteria, guidelines and protocols and to consider how to strengthen certain aspects, such as discharge criteria. Decisions on discharging vulnerable babies from any health facility at 1500 g, and even at 1800 g, require good clinical judgement. A thorough reflection on how to best reorganise and train the workforce for newborn care to achieve the most effective implementation of the Family-Led Care model may also improve the chances of success.

Because of poor record-keeping prior to the project, a common problem in Malawi, and the short time frame for implementation, it was not possible to collect baseline data with a proper monitoring and evaluation system in place. In the process of implementing the model, record-keeping continued to improve so that by the end of the project, the perception existed that all babies were now counted (unpublished end-of-project evaluation report) and that there had been an increase in the newborn survival rate. The Every Preemie project may have contributed to the foundation for continued monitoring and evaluation of newborn care in the district through the implementation of the Family-Led Care model.

Professionals’ knowledge and skills regarding the care of preterm and LBW babies were similar for the two data periods, but were uneven across the field and not up to standard. Possible reasons include low levels of basic newborn care knowledge and skills of the participating trainees. Furthermore, the relevant tools may not have been sufficiently validated in the pilot, something that is difficult in a dynamic context of implementation where training and orientation have been revised on the go to accommodate new insights. These tools are available in English only because the designers of the Family-Led Care model assumed sufficient proficiency to complete questionnaires in English (perhaps incorrectly), as professional providers receive their pre-service education in English.

This study illustrated how difficult it is to adequately pretest training and materials over a short project cycle that also had to include implementation research. The implementation of Family-Led Care in Balaka district served as a pilot for the further rollout of Family-Led Care. As a result of this project, training and counselling materials have been adapted to address identified shortcomings and could be further adapted for other contexts (https://www.everypreemie.org/family-led-care-global/). Health workers’ demonstration that they had mastered the three counselling scenarios according to the Family-Led Care flipbook confirms the usefulness of the flipbook as a means of ensuring that standardised counselling takes place for families of preterm and LBW babies.

The activities reported in this article provide an overview of the implementation of the Family-Led Care model and the influence on the health workers involved. This study demonstrated how Family-Led Care could be integrated into existing district newborn care services and thereby increase the coverage and quality of care for preterm and LBW babies receiving KMC as a life-saving intervention. Future studies could focus on identifying contextual factors that explain possible facilitators and barriers to the optimal implementation of the Family-Led Care model or the sustainability of practices after the end of the implementation project.

## Conclusion

This implementation study aimed to not only to look at ‘what’ works in the Family-Led Care model but also to understand ‘how to make it work’ and ‘how best’ to package and scale up the model. In this article, we reported key factors in the implementation of the model in Balaka district, Malawi, namely, the leadership of the District Health Management Team, the comprehensive clinical training and subsequent orientation to the model, and the quality improvement activities based on the needs identified by providers (see Table 1). We also demonstrated how quality of care improved through better documentation and better follow-up of preterm and LBW babies and how health workers made the Family-Led Care model work. Overall, health workers were positive about their involvement, and they reported positive reactions from families. This study also informed the development of a more universal Family-Led Care model to improve the care of preterm and LBW babies.^21^

Family-Led Care is a model in the broader circle of family-centred care models, specifically designed to improve the care of preterm and LBW babies in resource-constrained contexts. Of special importance is the way in which the follow-up care is linked to community health structures. Other countries can learn from this model when implementing models of care for preterm and LBW newborns.
